# Novel mitochondrial complex I-inhibiting peptides restrain NADH dehydrogenase activity

**DOI:** 10.1038/s41598-019-50114-2

**Published:** 2019-09-23

**Authors:** Yao-Peng Xue, Mou-Chieh Kao, Chung-Yu Lan

**Affiliations:** 1 Institute of Molecular and Cellular Biology, National Tsing Hua University, No. 101, Section 2, Kuang-Fu Road, Hsinchu, 30013 Taiwan, ROC; 2 Institute of Molecular Medicine, National Tsing Hua University, No. 101, Section 2, Kuang-Fu Road, Hsinchu, 30013 Taiwan, ROC; 3 Department of Life Science, National Tsing Hua University, No. 101, Section 2, Kuang-Fu Road, Hsinchu, 30013 Taiwan, ROC

**Keywords:** Biotechnology, Antifungal agents

## Abstract

The emergence of drug-resistant fungal pathogens is becoming increasingly serious due to overuse of antifungals. Antimicrobial peptides have potent activity against a broad spectrum of pathogens, including fungi, and are considered a potential new class of antifungals. In this study, we examined the activities of the newly designed peptides P-113Du and P-113Tri, together with their parental peptide P-113, against the human fungal pathogen *Candida albicans*. The results showed that these peptides inhibit mitochondrial complex I, specifically NADH dehydrogenase, of the electron transport chain. Moreover, P-113Du and P-113Tri also block alternative NADH dehydrogenases. Currently, most inhibitors of the mitochondrial complex I are small molecules or artificially-designed antibodies. Here, we demonstrated novel functions of antimicrobial peptides in inhibiting the mitochondrial complex I of *C*. *albican*s, providing insight in the development of new antifungal agents.

## Introduction

Antifungal drug resistance is related to high mortality and increased treatment cost of invasive fungal diseases, and is emerging as a serious threat to public health. Because only a few classes of antifungals are currently available, developing new antifungal agents and therapeutic strategies is urgently needed. Antimicrobial peptides (AMPs) function as important components of host innate immunity and have a broad spectrum of activity against a wide range of microbial pathogens^1,2^. Therefore, AMPs are considered potential new antifungal agents^3^.

AMPs are found at most sites in the human body and include defensins, histatins, cathelicidins, dermcidins, and hepcidins^2^. Among them, histatin 5 (Hst5) is a potent member of the histatin AMP family with activity against *C*. *albicans*. P-113 is a 12-mer amino acid fragment of Hst5 that retains strong candidacidal activity and has no adverse effects in clinical trials^4–7^. In our previous study^8^, we described the increased antifungal activity of P-113 derivatives P-113Du and P-113Tri, which contain higher fractions of amphipathic α-helices and are more stable at high-salt and low-pH conditions than P-113^8,9^. Moreover, P-113Du and P-113Tri exhibit low cytotoxicity and show prominent antifungal activity against planktonic cells, biofilm cells and clinical isolates of *C*. *albicans* and non-*albicans Candida* spp^8^. Notably, the AMP-treated biofilm cells show a rough appearance with protuberances on their surfaces, and these protuberances are largely reduced in the presence of ascorbic acid^8^. Interestingly, as a scavenger of reactive oxygen species (ROS), ascorbic acid enhances the viability of biofilm cells with AMP treatment^8^. These results imply that the generation of ROS  is correlated with the *C*. *albican*s killing by P-113Du and P-113Tri. However, the detailed mechanisms of this ROS-mediated candidacidal activity of P-113Du and P-113Tri are still not fully understood.

Mitochondria generate energy for cells through the oxidative phosphorylation system (OXPHOS). Mitochondrial complex I is the largest enzyme complex in OXPHOS and is the entry point for electrons into the respiration chain. It oxidizes NADH from the tricarboxylic acid cycle, reduces ubiquinone, and transports protons across the inner mitochondrial membrane, contributing to the generation of the proton-motive force (PMF) that is used to make ATP. Moreover, complex I is also a major generator for intracellular ROS. In this study, we demonstrated that P-113Du and P-113Tri, along with P-113, target *C*. *albicans* mitochondria by inhibiting complex I, particularly the NADH dehydrogenase subunit of the complex. Moreover, P-113Du and P-113Tri even exert their inhibitory functions against a fungus-specific alternative NADH dehydrogenases, and this inhibitory spectrum is rarely observed for complex I-specific inhibitiors^10^. Together, these findings provide new insights into antifungal mechanisms of AMPs.

## Results

### P-113Du and P-113Tri target mitochondria

**Figure 1:**
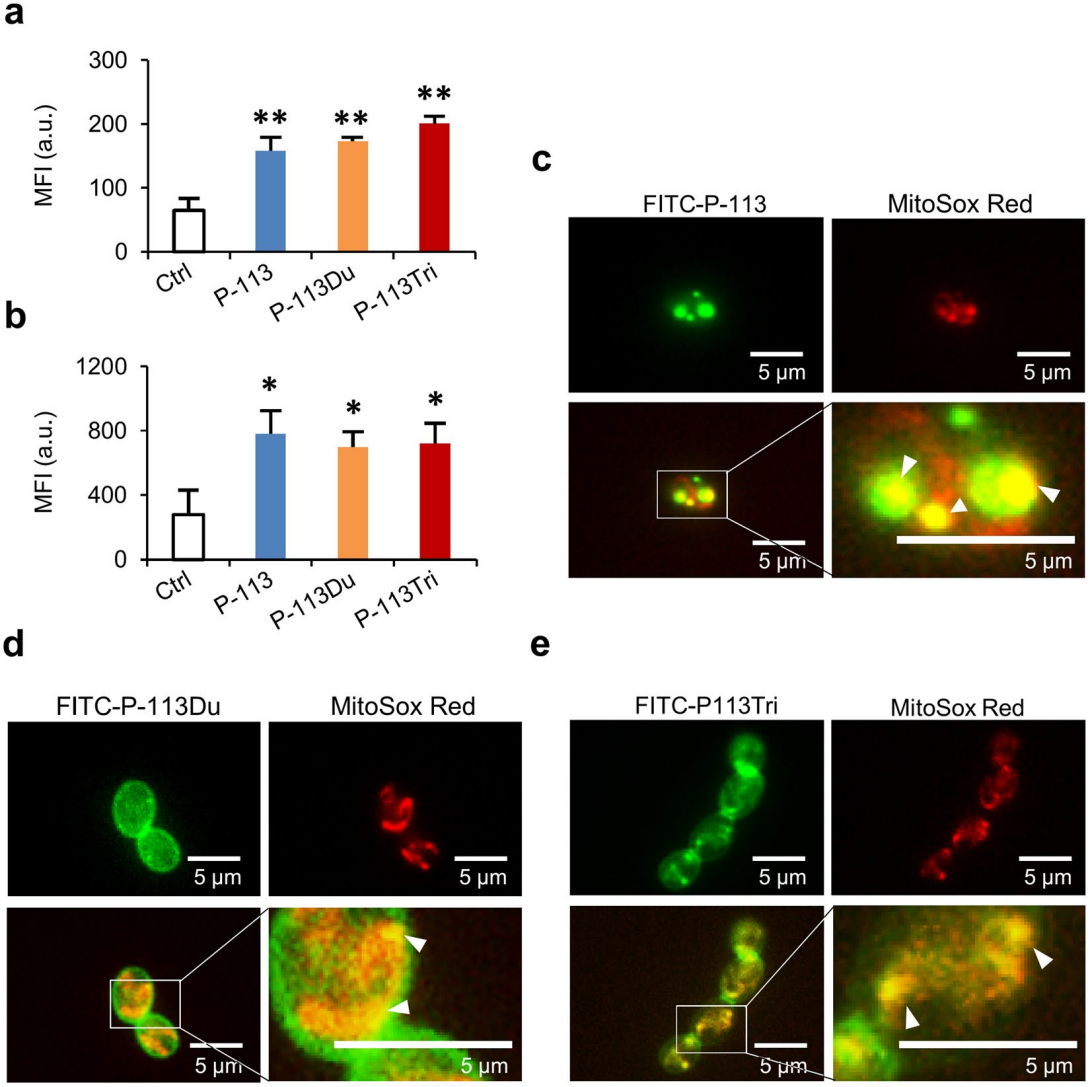
P-113, P-113Du and P-113Tri target mitochondria. The intracellular ROS levels were measured using the fluorescent dye H_2_DCFDA (**a**) and MitoSOX Red (**b**) after the cells were treated with a sublethal dose of the AMPs at 37 °C for 1 h. The results are represented as the mean ± SD of three independent experiments. P-values are derived from the cells treated with AMPs compared to the control cells without treatment. *P < 0.05; **P < 0.01. Fluorescent images of *C. albicans* with FITC (green)-labeled P-113 (**c**), P-113Du (**d**), and P-113Tri (**e**). Mitochondria were stained with MitoSox Red (red). The white triangles point to the colocalization of AMPs and mitochondria. Images of cells without AMP treatment are provided in Supplementary Fig. 2.

Our previous study suggested that the candidacidal activity of P-113Du and P-113Tri may be related to the generation of ROS^8^. To test this hypothesis, we first measured the level of intracellular ROS. In Fig. 1a, AMP-treated cells stained with the dye H_2_DCFDA showed higher fluorescence compared to the control cells (without AMP treatment). This result reflects an overall increased level of intracellular ROS in *C. albicans* cells with AMP treatment (Fig. 1a and Supplementary Fig. S1a). Moreover, the level of intracellular ROS was also measured using the MitoSox Red dye, which is an indicator of superoxide in the mitochondria of living cells. AMP-treated cells had a higher level of MitoSox Red fluorescence compared to that of control cells without AMP treatment (Fig. 1b and Supplementary Fig. S1b).

To further investigate the subcellular location of the peptides, FITC-labeled AMPs were used. The mitochondria of *C. albicans* were first visualized with MitoSox Red followed by the addition of FITC-labeled AMPs. The fluorescence microscopy images showed that the mitochondria of the control cells (without AMP treatment) was only stained with the MitoSox Red (Supplementary Fig. S2). However, the FITC-labeled P-113 mainly accumulated in the cytosol and colocalized with mitochondria (Fig. 1c). The FITC-labeled P-113Du and P-113Tri, on the other hand, seemed to localize on the cell surface, including the septum during cell division, and also colocalized with mitochondria (Fig. 1d,e). Together, our studies suggest that all of the AMPs tested can target mitochondria to induce ROS production.

### The candidacidal activity of P-113Du and P-113Tri is associated with mitochondrial respiration

**Figure 2:**
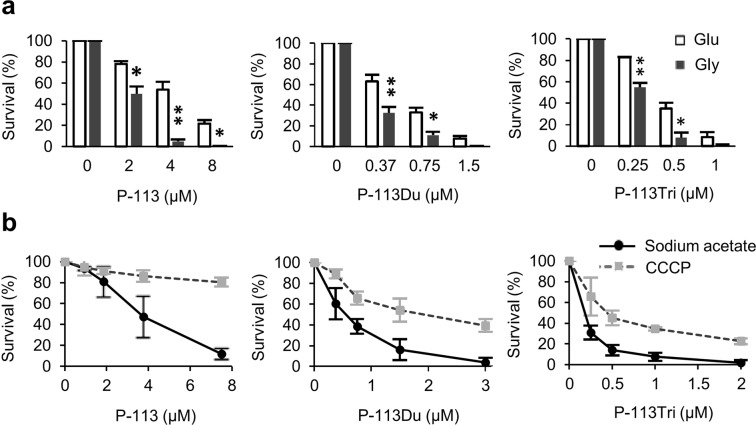
The AMPs affect cellular respiration. (**a**) Cells were grown in glycerol (Gly, 2% w/v) or glucose (Glu, 2% w/v) for 5 h, followed by the addition of AMPs and further incubation at 37 °C for 1 h. *P < 0.05; **P < 0.01 for cells grown in Glu vs. Gly. (**b**) Cells were grown at 37 °C for 1 h in the presence of AMP alone (dissolved in sodium acetate) or AMP + CCCP. Cell viability was determined by measuring colony-forming units that were normalized to the control without AMP treatment (as 100%). The results are represented as the mean ± SD of three independent experiments.

Mitochondria function as the powerhouse of cells by utilizing metabolites of carbohydrates to produce ATP. Carbohydrates go through glycolysis in the cytosol and the tricarboxylic acid cycle in the mitochondrial matrix to generate NADH and FADH_2_, which are then fed into the electron transport chain. Aerobic growth in nonfermentable carbon sources forces *C. albicans* cells to use mitochondrial oxidative phosphorylation for ATP production. Once mitochondrial respiratory stress is induced, cells grown in a nonfermentable carbon source, such as glycerol, are assumed to be more susceptible to AMPs. As shown in Fig. 2a, cells grown in glycerol exhibited a higher susceptibility to AMPs than cells grown in glucose, confirming the involvement of respiration in the actions of the tested AMPs.

To further verify the association between the peptides and mitochondrial respiration, susceptibility to carbonyl cyanide m-chlorophenyl hydrazine (CCCP) was assessed in cells cotreated with CCCP and AMPs. CCCP inhibits ATP production by disrupting the coupling of the electron transport chain with oxidative phosphorylation via neutralization of the proton gradient between the mitochondrial intermembrane space and the matrix. Therefore, under aerobic conditions, the CCCP-treated *C. albicans* cells perform fermentation and produce more ethanol than cells without CCCP treatment^11^. Indeed, our results indicated that cells treated with CCCP displayed a lower susceptibility to AMPs compared with the control cells without AMP treatment (Fig. 2b). Therefore, the candidacidal activity of P-113, P-113Du and P-113Tri is closely related to mitochondrial respiration.

### *C*. *albicans* mutants defective in mitochondrial complex I are resistant to P-113

**Figure 3:**
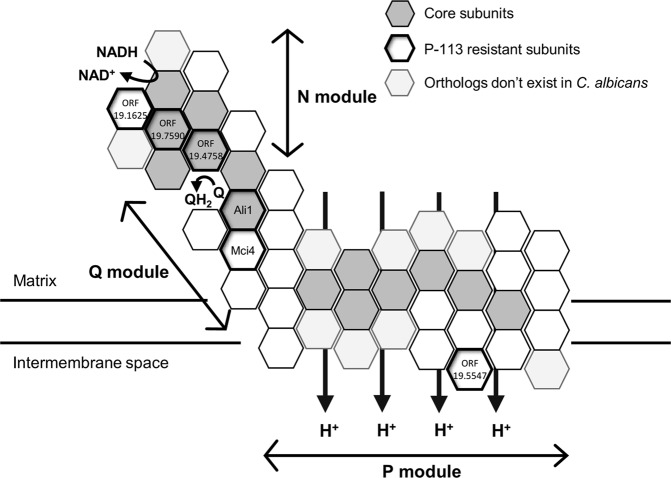
*C*. *albicans* mitochondrial complex I subunits are demonstrated. The possible distribution of core subunits in mitochondrial complex I is shown in gray blocks. Mutants resistant to P-113 are also indicated (outlined in bold). ORF19.7590, ORF19.1710 (Ali1) and ORF19.4758 appear to be core subunits that are conserved from bacteria to mammalian cells. ORF19.1625, ORF19.2570 (Mci4) and ORF19.5547 appear to be accessary subunits of mitochondrial complex I. These proteins, which are possibly targeted by P-113, are mostly components of the hydrophilic domain of complex I. This diagram is drawn based on the structural models of mitochondrial complex I from She *et al*.^14^.

To identify potential targets that may be attacked by the AMPs, we also screened a *C. albicans* homozygous mutant library^12^ by growing *C*. *albicans* cells in YPD medium with or without P-113 (100 μM). The results showed that the control SC5314 and SN250 cells were sensitive to P-113, whereas several mutant strains were resistant to P-113 (Supplementary Fig. S3). Interestingly, a subset of the P-113-resistant mutants lacked genes related to mitochondrial complex I of the electron transport chain (Table 1)^13–15^. Moreover, we used the BLAST search tool to identify matches of this subset of genes in *Yarrowia lipolytica*, a model yeast for mitochondrial study and in humans (*Homo sapiens*). We found that ORF19.287 (*NUO2*) and ORF19.2821 had no orthologs in *Y*. *lipolytica* or humans (Table 1). In addition, as shown in a simple model (Fig. 3), ORF19.7590, ORF19.1710 (*ALI1*) and ORF19.4758 appeared to be core subunits that are conserved from bacteria to mammalian cells, and ORF19.1625, ORF19.2570 (*MCI4*) and ORF19.5547 appear to be accessary subunits of mitochondrial complex I. These proteins are mostly considered to be components of the hydrophilic domain of respiratory complex I^14^.

**Table 1:** *C*. *albicans* mutants lacking mitochondrial complex I-related genes resistant to P-113 are identified by mutant library screening.

Systematic name	Gene name	Yarrowia lipolytica^a^	Homo sapiens^a^
ORF19.7590 ^b^		NUAM	NDUFS1
ORF19.287	NUO2	N/A	N/A
ORF19.1625		N7BM	NDUFA12
ORF19.1710 ^b^	ALI1	NUGM	NDUFS3
ORF19.2570	MCI4	NUFM	NDUFA5
ORF19.2821		N/A	N/A
ORF19.4758 ^b^		NUIM	NDUFS8
ORF19.5547		NI2M	NDUFB9

^a^The orthologs of *C*. *albicans* genes in *Y*. *lipolytica* and *H*. *sapiens*.

^b^Conserved core subunits in all species having complex I are underlined.

### The AMP treatment inhibits cell respiration in *C. albicans*

**Figure 4:**
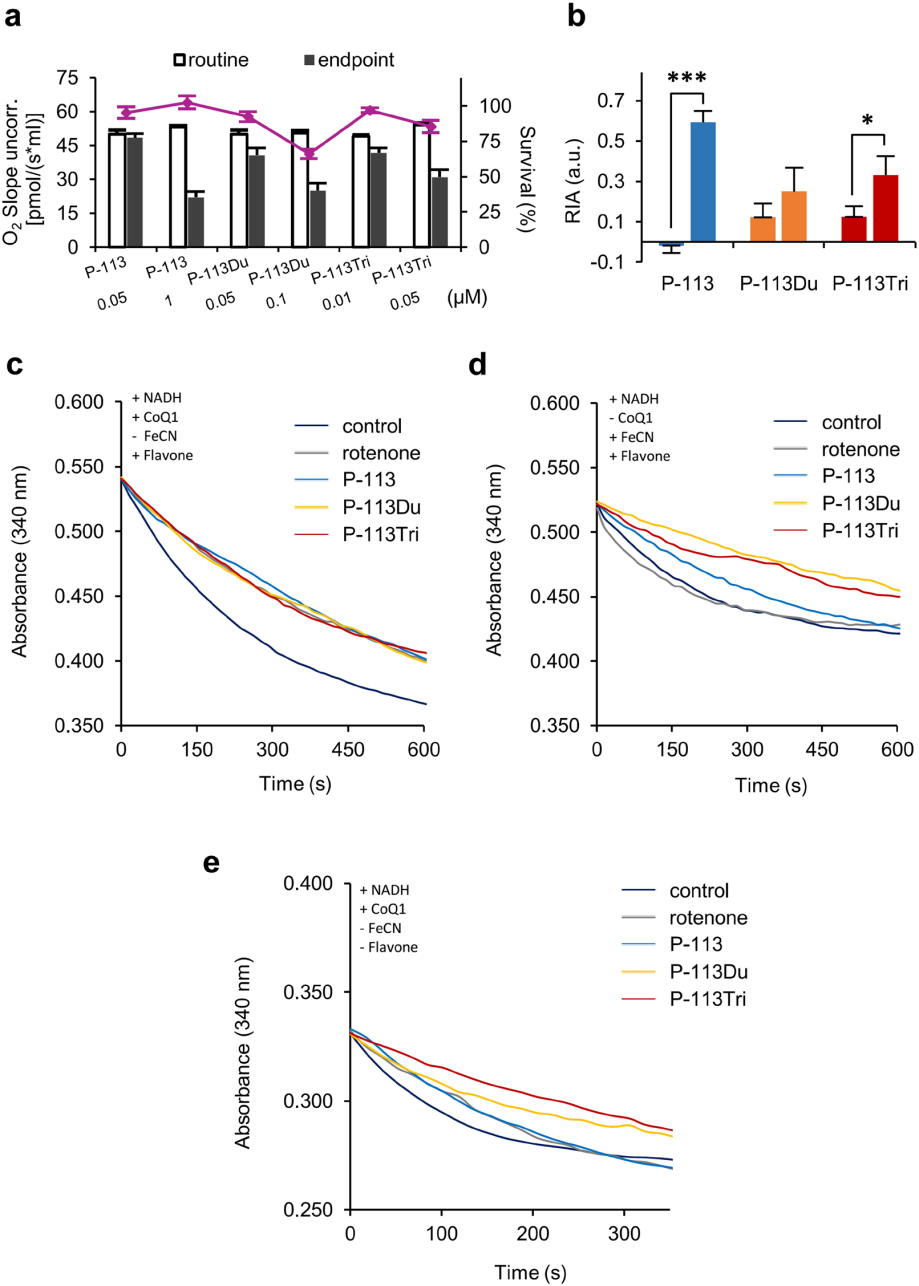
The AMPs effectively inhibit respiration and NADH dehydrogenases. (**a**) The oxygen consumption rates and survival rates (purple line) of cells treated with AMPs are shown. The survival rates were determined by recording the number of colony-forming units from the cells used for the oxygen consumption assays. The white bar represents the routine respiration rate of the cells and the black bar represents the endpoint of the respiration rate after AMP addition. The results are presented as the mean ± SD of three independent experiments. (**b**) The relative inhibitory activity (RIA) was determined by normalization of the inhibitory rate to the survival rate of the AMP-treated cells. The AMP concentrations from left to right are P-113 (0.05 μM), P-113 (1 μM), P-113Du (0.05 μM), P-113Du (0.1 μM), P-113Tri (0.01 μM), P-113Tri (0.05 μM). (**c**) Mitochondria were isolated from *C. albicans* and treated with rotenone or various AMPs in the presence of flavone with CoQ1 as the electron acceptor. NADH consumption was determined by measuring the absorbance at 340 nm. (**d**) The inhibitory effect of AMPs on NADH oxidization by complex I was assayed using ferricyanide instead of CoQ1 as the electron acceptor. (**e**) The effects of AMPs on alternative NADH dehydrogenases using an assay similar to (**c**) but without the addition of flavone. For (**c**) to (**e**), all experiments were repeated independently at least three times and the representative plots from three experiments with similar results are shown.

Mitochondrial complex I is a multisubunit complex related to cellular respiration and oxygen consumption. Therefore, to further evaluate the connection between the peptides and mitochondrial complex I of the respiration chain, the respiratory oxygen consumption rate of *C. albicans* was measured dynamically using an Oxygraph-2k respirometer. Notably, the oxygen consumption rate was significantly decreased in cells following AMP treatment compared to the control cells without AMP treatment (Fig. 4a, Supplementary Fig. S4). These results support the notion that P-113Du, P-113Tri and P-113 have an activity that inhibits cellular respiration in *C. albicans*. Furthermore, cells treated with P-113Tri and P-113Du displayed relatively gentle slopes as they approached their endpoint oxygen consumption rate compared to cells treated with P-113 (Supplementary Fig. S4b–S4d). This result implied that P-113Tri and P-113Du may need more time to exert their activity than P-113. To further understand the efficiency of the AMPs to inhibit respiration, the relative inhibitory activity (RIA) was determined by normalization of the inhibitory rate to the survival rate of the AMP-treated cells (Fig. 4b). Using the same concentrations, the results show that P-113Tri has the highest inhibitory activity on cellular respiration, followed by P-113Du and P-113.

### NADH dehydrogenase of mitochondrial complex I is inhibited by the AMPs

The electron transport chain in mitochondrial complex I is initiated by NADH oxidization, followed by electron transfer to ubiquinone (coenzyme Q10, CoQ10). To continue to assess the effects of P-113, P-113Du and P-113Tri on mitochondrial complex I, mitochondria were isolated and treated with different concentrations of the AMPs in the presence of NADH and CoQ1, a soluble CoQ analog. In this reaction, mitochondrial complex I consumes NADH and reduces CoQ1. The NADH consumption rate was examined by measuring the absorbance at 340 nm, and the complex I activity was presented as the slopes of lines in a plot of the NADH consumption^16^. In addition to complex I, yeasts commonly contain other types of NADH dehydrogenases, called alternative NADH dehydrogenases, in their mitochondria^17^. In the study of *Saccharomyces cerevisiae*, the internal alternative NADH:ubiquinone oxidoreductase (Ndi1) is also able to oxidize NADH within mitochondrial matrix and feeds electrons into the respiratory chain^18^. Therefore, to reduce possible noise caused by *C. albicans* alternative NADH dehydrogenases in the assay, flavone, a specific rotenone-insensitive internal NADH dehydrogenase inhibitor, was introduced to decrease the compensatory effect of noncomplex I NADH consumption^18,19^. Our experiment revealed that P-113, P-113Du and P-113Tri all affect the NADH consumption rate in the presence of flavone, and this result was similar to that of mitochondria treated with rotenone, a well-known mitochondrial complex I inhibitor^20^ (Fig. 4c). However, mitochondria without any treatment showed a very rapid NADH consumption rate (Fig. 4c). Moreover, the inhibitory activity of the AMPs against complex I is dose-dependent (Supplementary Fig. S5a–S5c).

Mitochondrial complex I is also called NADH: quinone oxidoreductase as it contains hydrophilic domain consisting of NADH dehydrogenase and ubiquinone reductase. Ferricyanide is a chemical that reacts with NADH dehydrogenase, causing the electrons to bypass ubiquinone and to be directly transferred to ferricyanide. In other words, ferricyanide leads to rotenone-insensitive NADH oxidation^21^. Therefore, by replacing CoQ1 with ferricyanide, it should be possible to detect the specific activity of NADH dehydrogenase in complex I. In Fig. 4d, we found that rotenone lost its activity to inhibit complex I in the presence of ferricyanide. However, P-113Du and P-113Tri as well as P-113 still retained their inhibitory activity against complex I NADH dehydrogenase, especially P-113Tri and P-113Du (Fig. 4d). These actions of the AMPs are correlated with the concentrations of peptides used (Supplementary Fig. S5d–S5f).

The yeast *S. cerevisiae* has no mitochondrial complex I. However, alternative NADH dehydrogenases, such as Nde1 and Ndi1, can perform similar functions as complex I, facilitating NADH oxidation for the electron transport chain^22^. Orthologs of these alternative NADH dehydrogenases exist in *C. albicans* and are annotated as Nde1 and Ymx6^19^, respectively. In *C. albicans*, Nde1 and Ymx6 can compensate for the function of defective mitochondrial complex I^19^. To examine the possible activity of the AMPs against alternative NADH dehydrogenases, we removed flavone from the assay and used CoQ1 as the electron acceptor. Mitochondria treated with rotenone alone showed a partial inhibition of NADH consumption compared to the control without rotenone treatment (Fig. 4e). The P-113-treated mitochondria exhibited a similar rate of NADH consumption compared to that of the rotenone-treated mitochondria. In contrast, mitochondria treated with both P-113Du and P-113Tri exhibited slower NADH consumption rates (Fig. 4e). These results suggest that P-113 inhibits *C. albicans* complex I but not the alternative NADH dehydrogenases. However, P-113Du and P-113Tri are broad spectrum inhibitors of NADH dehydrogenases, including that of complex I and the alternative NADH dehydrogenases.

### The killing mechanism of the AMPs is closely correlated with intracellular ROS generation

**Figure 5:**
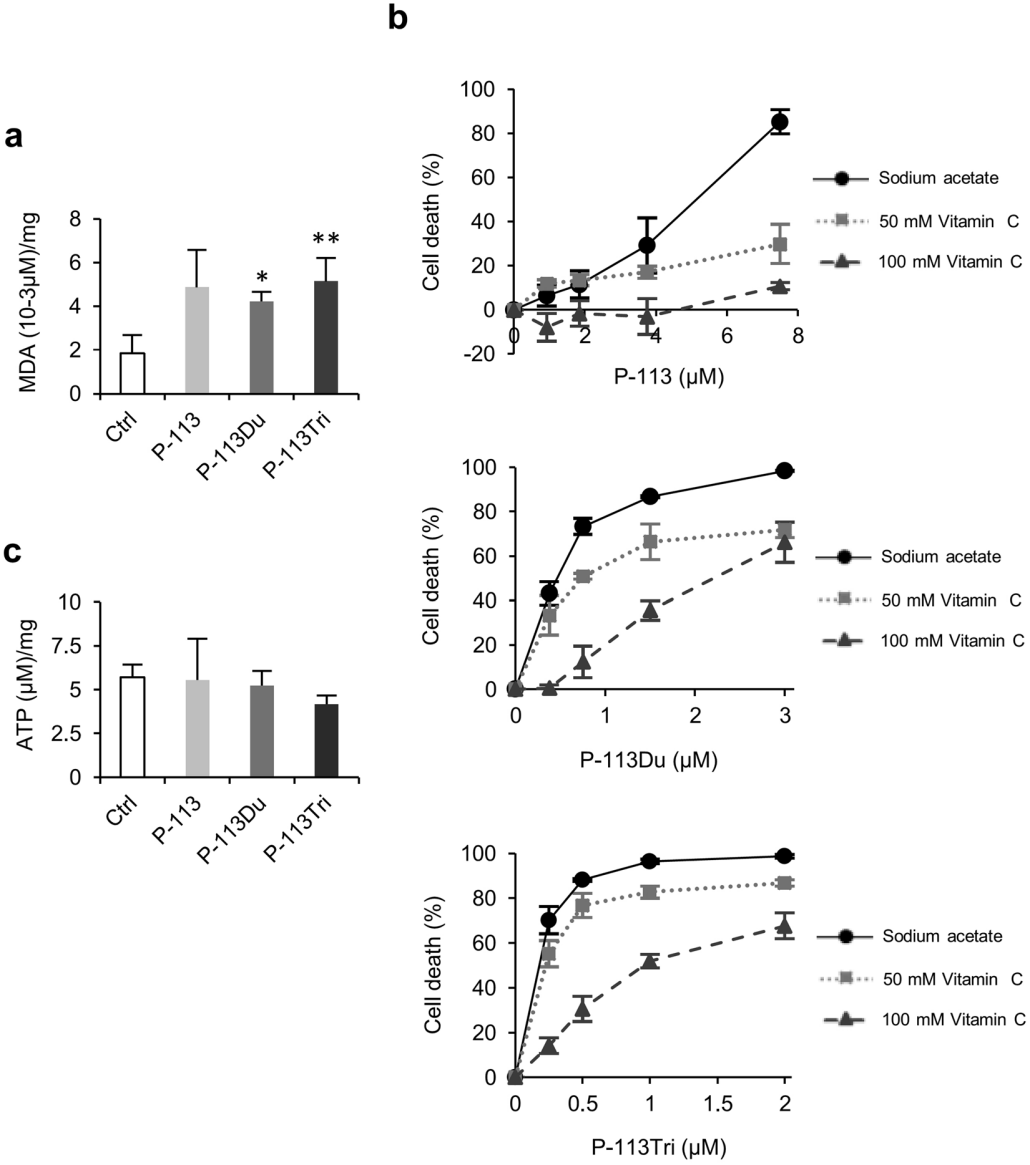
ROS induction is associated with the candidacidal activity of AMPs. (**a**) The levels of malondialdehyde, a lipid peroxidation marker, were measured at 530 nm in cells treated with or without AMPs for 2 h. The MDA content was calculated and expressed as 10^−3^ μM/mg protein. *P < 0.05; **P < 0.01 for cells with AMP treatment vs. the control (without AMP treatment). (**b**) The ROS scavenger ascorbic acid reduces cellular susceptibility to AMPs. *C. albicans* cells were treated with AMPs, incubated at 37 °C for 1 h, plated on YPD agar and the colony-forming units (cfu) were counted. The number of cfus of the AMP-treated cells were normalized to the control cells without treatment. (**c**) The intracellular ATP content of AMP-treated *C*. *albicans* cells was measured using a luciferase-based assay. Luminescence was measured at 560 nm. ATP content is shown as (μM)/mg protein. The results are represented as the mean ± SD of three independent experiments.

Blocked cellular respiration increases the level of reducing flavin and the NADH/NAD^+^ ratio, causing overproduction of O_2_^−^ and other free radicals^23,24^. These oxidative stressors damage macromolecules such as nucleic acids, proteins, and lipids. For example, lipid peroxidation occurs when ROS attack the double bond of unsaturated fatty acids to trigger a chain reaction to generate more free radicals^25^. Our findings above showed that cells treated with P-113, P-113Du and P-113Tri have disrupted mitochondrial complex I activity and exhibit an increase in intracellular ROS levels (Fig. 1a,b). Therefore, it is interesting to further assess the effects of ROS on the cells. Figure 5a shows an elevation in the malondialdehyde (MDA) contents in cells treated with P-113, P-113Du and P-113Tri. MDA is formed during lipid peroxidation and is an important marker for oxidative stress.

To further verify the effects of ROS on cell damage, the ROS scavenger ascorbic acid^26,27^, was used. Cells were treated with the AMPs in the presence or absence of ascorbic acid. The results indicated that ascorbic acid can effectively reduce the susceptibility of the cells to the AMPs (Fig. 5b). In particular, a high concentration of ascorbic acid (100 mM) can rescue almost all of the P-113-treated cells. This result is consistent with our previous study showing that an ROS scavenger can rescue biofilm cells treated with the AMPs^8^.

Inhibition of complex I may not only kill the cells by inducing ROS, but may also decrease the ATP level of the cells due to the interruption of mitochondrial respiration. We measured the ATP content of cells with AMP treatment for 1 h. The results showed that there was no significant difference in the ATP content between the AMP-treated cells and the control cells without AMP treatment (Fig. 5c). Therefore, the rapid-killing by AMPs does not involve the depletion of energy, but rather occurs mostly through the induction of ROS generation.

## Discussion

Antimicrobial peptides (AMPs) are potential candidates for developing new antifungal drugs^3,28^. P-113 is an AMP originally derived from human salivary histatin 5 that exhibits potent activity against *C*. *albicans*^4,29^. *C*. *albicans* mutants defective in respiration are shown to be Hst5 resistant^30^, and Hst5 likely targets mitochondria^31^. Enhanced ROS production and reduced oxygen consumption rate^32^ are also reported in cells treated with Hst5. The study by Petruzzelli and colleagues showed that Hst5 is unable to pass through the cell membrane of mammalian cells^33^. However, in the presence of mitochondrial complex I-linked substrates, glutamate-malate, Hst5 could inhibit respiration in isolated mitochondria, suggesting that it targets mitochondrial complex I.

Although P-113 is originally derived from Hst5, the mode of action of P-113 is relatively less studied. Notably, the efficacy of P-113 is significantly reduced under high-salt and low-pH conditions^8^. In our previous study, the synthetic AMPs P-113Du and P-113Tri were designed based on P-113, and they show low levels of hemolytic and toxic effects in mammalian cells^8^. Compared to P-113, these AMPs are more tolerant to high salt and low-pH and exhibit an increased activity against planktonic cells, biofilm cells and clinical *Candida* isolates^8^.

In this study, we further studied the mechanisms of action for P-113Du and P-113Tri along with P-113 and found that these AMPs inhibit respiration by targeting the NADH dehydrogenase in mitochondrial complex I. To date, mitochondrial complex I inhibitors are mostly small molecules^10,34^ or artificially-designed antibodies^35^. These small molecular inhibitors usually contain a cyclic structure that is similar to the ubiquinone ring^10,36^, resulting in their specific function of blocking electron transfer on ubiquinone reductase of complex I. The NADH dehydrogenase subunit of complex I, on the other hand, is an unusual protein complex with a limited repertoire of available inhibitors^21,37–39^. Natural analogues are the most common sources for inhibitors, which can compete with the original substrate of NADH dehydrogenases. However, neither adenosine, nicotinamide mononucleotide nor nicotinamide adenine dinucleotide (NAD^+^, the closet analogue of NADH) could be used as NADH oxidation inhibitors^37^. Interestingly, P-113Du and P-113Tri inhibit not only complex I NADH dehydrogenases but also alternative NADH dehydrogenases present in mitochondria. To our knowledge, this is the first report showing these special features from AMPs.

In *C. albicans* pathogenicity, energy exploitation is usually related to the availability of nutrients, such as glucose. However, glucose is limited in different niches within the human host^40,41^, and *C. albicans* facilitates metabolic adaptation by upregulating gluconeogenesis, the glyoxylate cycle, and fatty acid β-oxidation^40^, which all require the participation of mitochondria. Therefore, disrupting the function of mitochondria may provide a good strategy against *C. albicans* and other fungal pathogens.

In addition to metabolism, mitochondria also contribute to the *C. albicans* stress response, antifungal drug tolerance, cell wall biosynthesis and structure, yeast-hyphae morphogenesis, biofilm formation and immune interaction^15,42–45^. For example, a biofilm is a complex 3D structure of surface-attached microbial communities. *C*. *albicans* biofilm cells are more resistant to antimicrobial agents (1000 times more resistant in some cases), compared to the planktonic cells^46^. Hyphal morphogenesis is a critical process required for *C. albicans* to form a mature biofilm^46^. Moreover, the planktonic yeast cells are believed to be involved in dissemination during infections, whereas hyphal cells contribute to host tissue invasion and damage^47^. A hypothesis proposed by Calderone, Traven and colleagues is that mitochondria are required during the early stage of biofilm formation, including hyphal development, and that mitochondrial function may gradually decline due to the availability of nutrients and oxygen^45^. Gene expression profiling and metabolomics analysis suggest the downregulation of mitochondrial activity in biofilms; however, mitochondrial respiration is required for both *C*. *albicans* morphogenesis and consequently for biofilm maturation^45^. Importantly, our previous study demonstrated P-113Du and P-113Tri as well as P-113 can eradicate up to 90% of biofilm cells^8^. Moreover, several respiratory-deficient mutants appear to be resistant to P-113 (Table 1), and respiratory-deficient *C*. *albicans* mutants have attenuated virulence^42,44,48,49^. Together, these results demonstrate the importance of mitochondria in *C. albicans* physiology and virulence, further supporting mitochondria as potential targets for the development of new antifungal agents^41,42^.

In this work, we also determined the localization of the AMPs within *C. albicans* cells. P-113 is mainly observed within *C*. *albicans* cells. While, P-113Du and P-113Tri appear to not only gain intracellular access but also to associate with the cell surface and septum (Fig. 1c–e). The higher fungicidal activity of P-113Du and P-113Tri compared to that of P-113 may also relate to their dual cell surface/mitochondria localization. However, the biology underlying these observations for both P-113Du and P-113Tri, such as their inhibitory activity against alternative NADH dehydrogenases and the interaction with the *C*. *albicans* cell surface and septum, must be further investigated.

In summary, our previous study showed that P-113Du, P-113Tri and P-113 exhibited candidacidal activity against biofilm cells, although the activity varied among these AMPs^8^. Here, we further demonstrated that these AMPs target mitochondria, halt respiration and kill *C*. *albicans* cells through the induction of ROS generation (Figs 1a,b, 5). Although the detailed functions of P-113, P-113Du, and P-113Tri in the destruction of *C*. *albicans* biofilms remain to be determined, considering our findings in combination with those of other studies reveals a complex interplay between mitochondria, physiology and virulence in *C. albicans*. Moreover, we identified several *C. albicans* mitochondrial complex I components that had no orthologs in humans, as potential targets of AMPs (Table 1 and Fig. 3). Together, our results suggest the potential use of AMPs in therapeutic applications and highlight the complex mechanisms and functions that may be at play during AMP-mediated killing.

## Methods

### Peptides and reagents

P-113 (AKRHHGYKRKFH-NH_2_), P-113Du (AKRHHGYKRKFHAKRHHGYKRKFH-NH_2_), P-113Tri (AKRHHGYKRKFHAKRHHGYKRKFHAKRHHGYKRKFH-NH_2_) and FITC-labeled peptides were synthesized by MDBio, Inc. (Taipei, Taiwan). HPLC and mass spectrometry showed the purity of the peptides was >95%. The peptides were dissolved in 12.5 mM sodium acetate (pH 6.0)^8^. All reagents used were obtained from Sigma-Aldrich (St. Louis, MO) unless stated otherwise.

### Strains, media and growth condition

*C. albicans* SC5314 was used throughout this study. Cells were maintained at −80 °C and plated on YPD agar: 1% (w/v) yeast extract, 2% (w/v) peptone, 2% (w/v) glucose and 1.5% (w/v) agar, before each experiment. A single colony was inoculated into YPD broth and grown at 30 °C overnight (~16 h) with shaking at 180 rpm. The overnight culture was harvested by centrifugation and cell pellets were subcultured (for ~5 h) in YPD or synthetic complete (SC) medium (0.67% (w/v) yeast nitrogen base [YNB] without amino acids, 0.079% (w/v) complete supplement mixture with 2% (w/v) glucose or 2% (w/v) glycerol). Glycerol was purchased from AMRESCO (Solon, OH).

### Isolation of mitochondria

Mitochondria were isolated as previously described^22^ with some modifications. Briefly, *C. albicans* cells were harvested by centrifugation, washed twice with deionized water and incubated in 1 M Tris-HCl (pH 9.3) and 10 mM dithiothreitol at 30 °C for 20 min. Cell pellets were then collected by centrifugation and incubated in buffer I (1.2 M sorbitol and 20 mM potassium phosphate) containing 2 mg/ml zymolase (AMSBIO, Oxfordshire, UK) at 30 °C for 1 h. Subsequent steps were performed at 4 °C or on ice. After centrifugation, spheroplasts were collected, washed twice with buffer I and resuspended in buffer II (0.6 M sorbitol, 10 mM Tris-HCl and 1 mM EDTA, pH 7.4). Cells were homogenized with 20 strokes by a hand-held glass homogenizer (CFA glass, Hsinchu City, Taiwan) and the supernatant was diluted twofold with buffer II. The supernatant was collected by centrifugation (5 min, 1500 × g) and spun again (5 min, 4000 × g). Finally, the samples were centrifuged (15 min, 12000 × g) and the resulting pellets with mitochondria were resuspended in buffer III (250 mM sucrose and 10 mM MOPS-KOH, pH 7.2) and kept on ice.

### Mitochondrial complex I activity assay

Mitochondrial complex I activity was determined by measuring the oxidation of NADH as described previously^16^ with some modifications. Briefly, the protein concentrations of the mitochondrial preparations were determined by Bradford protein assays (Bio-Rad) and the mitochondria were diluted to 500 μg protein/ml in air-saturated buffer III. AMPs (100 μM, 20 μM, 4 μM) and rotenone (100 μM) were prepared with buffer III. The mitochondrial solution (25 μl) and AMPs (or rotenone; 25 μl) were mixed in each well of a 96-well microplate with aerobic incubation at 37 °C for 2 h. Subsequently, 20 μl of AMPs- or rotenone-treated mitochondria (50 μg protein/ml) was transferred to each well of a microplate and mixed with 80 μl of assay buffer. The mitochondrial complex I activity was measured immediately at 340 nm using a VICTOR3 multilabel reader (Perkin Elmer). The assay buffer was prepared fresh and made of a hypotonic buffer (KH_2_PO_4_ 25 mM, MgCl_2_ 5 mM, pH 7.4), supplemented with 5 mg/ml BSA, 2 mM KCN, 2 μg/ml antimycin A (AA), 350 μM NADH, and 100 μM coenzyme Q1 (CoQ1), with or without 200 μM flavone. Mitochondria without AMPs or rotenone treatment were used as positive controls. All experiments were repeated independently at least three times.

### Identification of P-113-resistant mutants

To screen for novel targets affected by the P-113 AMP, the *C. albicans* homozygous deletion mutant library from Noble *et al*.^12^ was used. Mutants and the wild-type reference strain SN250 from the library were grown as previously mentioned, diluted with 12.5 mM sodium acetate to 8 × 10^6^ cells/ml, and treated with or without 100 μM of P-113 at 37 °C for 1 h as previously described^4^. Thereafter, 50 μl of cells were spotted onto YPD agar and incubated at 30 °C for 24 h. Cell growth was monitored and compared between each strain with or without P-113 treatment. The mutants screened were further verified by repeating the assay once. Orthologs of the potential target genes revealed in this mutant library screen were identified in *Yarrowia lipolytica* and *Homo sapiens* using BLAST (https://blast.ncbi.nlm.nih.gov/Blast.cgi).

### Antifungal susceptibility assay

The susceptibility of *C. albicans* to AMPs was determined using twofold serial dilutions of AMPs in sodium acetate (12.5 mM), ranging from 16 μM to 1 μM. Cells from overnight culture were harvested and subcultured in YPD and grown to exponential phase. Cells were collected by centrifugation, washed twice with 12.5 mM sodium acetate and resuspended in 12.5 mM sodium acetate to 8 × 10^6^ cells/ml. *C. albicans* cells and AMPs (50 μl each) were mixed and incubated at 37 °C for 1 h. Samples were spotted onto YPD agar plates. After incubated at 30 °C for 24 h, cell viability was accessed by counting colony forming units (cfu).

To assess the antifungal activity of the AMPs in different carbon sources, cells were grown overnight in YPD, subcultured in SC medium with 2% (w/v) glucose or 2% (w/v) glycerol and grown to exponential phase. The cell pellets were collected and treated with AMPs as describe above. To determine the effects of carbonyl cyanide m-chlorophenyl hydrazine (CCCP) or ascorbic acid on the antifungal activity of the AMPs, AMPs were prepared and diluted as describe above except CCCP (5 μM) or ascorbic acid (50 mM, 100 mM) were included. Cells without AMP treatment were used as controls. Concentration-killing curves showing the killing rate were plotted after counting the cfus and normalizing to the control cells. All experiments were repeated at least three times.

### Fluorescence colocalization of AMPs

Cells were grown overnight, subcultured in YPD broth and grown to exponential phase. Cells were harvested by centrifugation and washed twice with phosphate-buffered saline (PBS). The cells were placed in PBS buffer containing MitoSOX Red (5 μM) and incubated at 30 °C for 30 min to stain the mitochondria. Subsequently, cells were washed twice with PBS, twice with 12.5 mM sodium acetate, and then resuspended in 12.5 mM sodium acetate to 2 × 10^6^ cells/ml. Cells were treated with different concentrations of FITC-labeled AMPs (P-113, 1 µM; P-113Du, 0.1 µM; P-113Tri, 0.05 µM). The localization of the AMPs was detected using a Zeiss Axio Imager A1 fluorescence microscope (HBO100 Mercury Lamps, Filter set 56 HE: EX (GFP) BP 470/27, EX (DsRed) BP 556/25, Zeiss n-achroplan 100×/1.25 oil lens). All images were taken within 1 h at 37 °C after AMP addition. A total of 168 images were taken and analyzed with the SPOT Basic software (SPOT Imaging, Sterling Heights, USA).

### Measurement of intracellular ROS accumulation

Intracellular ROS content was detected by two fluorescent probes, cell- permeable 2′, 7′-dichlorodihydrofluorescein diacetate (H_2_DCFDA) and MitoSOX Red. Briefly, cells were grown overnight, subcultured in YPD broth and grown to exponential phase. Cells were collected by centrifugation, washed twice with 12.5 mM sodium acetate and resuspended in 12.5 mM sodium acetate to 8 × 10^6^ cells/ml. Cells were treated with sublethal dose of AMPs (P-113, 2 µM; P-113Du, 0.2 µM; P-113Tri, 0.1 µM) and incubated at 37 °C for 1 h. Subsequently, cells were harvested, washed twice with PBS and resuspended in PBS containing H_2_DCFDA (20 μg/ml) or MitoSOX Red (5 μM). The mixture was incubated at 30 °C for 30 min. Cell pellets were then collected, washed three times with PBS, and resuspended in PBS and then kept on ice. The fluorescent intensity was determined using a flow cytometer (BD Accuri^TM^ C6) and data was analyzed using the BD Accuri C6 software (version: 1.0.264.21). Briefly, cell separation was based on FSC-A/SSC-A to discriminate between living cells and cell debris. The AMP-treated cells with a similar FSC-A/SSC-A to the untreated cells were selected in the experiments. Cells without AMP treatment were used as negative controls. All experiments were repeated at least three times and the average fluorescent intensity was determined.

### Measurement of *C. albicans* respiration

The respiration or oxygen consumption rate of *C. albicans* cells was measured at 37 °C with an Oxygraph-2k instrument (O2k, OROBOROS Instruments, Innsbruck, Austria). *C. albicans* cells from overnight culture were inoculated into YPD medium and grown to exponential phase. Cells were harvested and resuspended in 12.5 mM sodium acetate. The basal oxygen consumption level was adjusted to 50 pmol/(s*ml). Cells were then treated with AMPs (P-113: 1 μM, 0.05 μM; P-113Du: 0.1 μM, 0.05 μM; P-113Tri: 0.05 μM, 0.01 μM), and the oxygen consumption rate was recorded until the readings stabilized. After the measurement, cells were collected, spotted (50 μl) onto YPD agar plates, and incubated at 30 °C for 24 h to assess cell viability. The basal oxygen consumption rate of *C. albicans* was normalized to its survival rate. The respiratory inhibitory activity (RIA) of the AMPs was calculated using the following formula we created:

$${\rm{Respiratory}}\,{\rm{inhibitory}}\,{\rm{activity}}\,({\rm{a}}.{\rm{u}}.)=1-\frac{Endpoint\,{O}_{2}\,consumption\,rate}{Basal\,{O}_{2}\,consumption\,rate\times (\frac{ \% \,Survival\,rate}{100})}$$

The basal O_2_ consumption rate is the routine oxygen consumption rate of cells without any treatment. The endpoint of O_2_ consumption rate reflects the stable oxygen consumption rate of cells after AMP treatment. The basal oxygen consumption rate was normalized to the cellular survival rate for each independent run. All experiments were repeated at least three times.

### Measurement of malondialdehyde (MDA) in *C. albicans*

*C. albicans* cells from overnight culture were grown in YPD medium to exponential phase. Cells were harvested, washed twice with 12.5 mM sodium acetate and resuspended in 12.5 mM sodium acetate to 4 × 10^6^ cells/ml. A total of 100 ml of cells were treated with AMPs (P-113, 1 μM; P-113Du, 0.1 μM; P-113Tri, 0.05 μM) in 12.5 mM sodium acetate and incubated at 37 °C for 2 h. Subsequently, cells were harvested, washed twice with PBS and mixed with ice-cold protein extraction buffer (5 mM potassium phosphate, 0.9% (w/v) sodium chloride, and 0.1% (w/v) dextrose, pH 7.4) and 0.3 g acid-washed glass beads (Sigma-Aldrich, 425~600 μm). Cells were disrupted by vortexing for 20 s and immediately cooled on ice for 20 s. The process was repeated eight times. Cell lysates were centrifuged (13000 × g, 4 °C, 15 min) and the supernatants were collected. Total protein was quantified by Bradford protein assay at 600 nm using an iMark microplate absorbance reader (Bio-Rad). The MDA assay was performed using the TBARS (TCA method) Assay Kit (Cayman Chemical), following the manufacturer’s instruction. All experiments were repeated at least three times.

### Measurement of ATP content in *C. albicans*

*C. albicans* cells from overnight culture were subcultured in YPD and grown to exponential phase. Cells were collected by centrifugation, washed twice with 12.5 mM sodium acetate and resuspended in 12.5 mM sodium acetate to 8 × 10^6^ cells/ml. *C. albicans* cells were treated with AMPs (P-113, 2 μM; P-113Du, 0.2 μM; P-113Tri, 0.1 μM) in 12.5 mM sodium acetate and incubated at 37 °C for 1 h. The supernatant containing ATP was collected using the protein extraction process mentioned above in the description of the MDA assay. The ATP level was evaluated using the luciferase assay in the ATP Determination Kit (Thermo Fisher), and luminescence was measured at 560 nm. All experiments were repeated at least three times.

### Statistical analysis

Two-tailed Student t-test was applied to assess the differences between samples. Differences were considered statistically significant for *p* < 0.05.

## Supplementary information

Supplementary Dataset

## Data Availability

The datasets generated during and/or analyzed during the current study are available from the corresponding author on reasonable request.

## References

[1] Bahar AA, Ren D (2013). Antimicrobial peptides. Pharmaceuticals (Basel).

[2] Wang G (2014). Human antimicrobial peptides and proteins. Pharmaceuticals (Basel).

[3] Ng SMS (2017). Antifungal peptides: a potential new class of antifungals for treating vulvovaginal candidiasis caused by fluconazole‐resistant Candida albicans. Journal of Peptide Science.

[4] Rothstein DM (2001). Anticandida activity is retained in P-113, a 12-amino-acid fragment of histatin 5. Antimicrob Agents Chemother.

[5] Driscoll J (1996). Candidacidal activity of human salivary histatin recombinant variants produced by site-directed mutagenesis. Gene.

[6] Helmerhorst EJ, Wim VTH, VEERMAN EC, Simoons-Smit I, Arie V (1997). Synthetic histatin analogues with broad-spectrum antimicrobial activity. Biochemical Journal.

[7] Mickels N (2001). Clinical and microbial evaluation of a histatin‐containing mouthrinse in humans with experimental gingivitis. Journal of clinical periodontology.

[8] Lin G-Y (2016). The Antimicrobial Peptides P-113Du and P-113Tri Function against Candida albicans. Antimicrob Agents Chemother.

[9] Jang WS, Li XS, Sun JN, Edgerton M (2008). The P-113 fragment of histatin 5 requires a specific peptide sequence for intracellular translocation in Candida albicans, which is independent of cell wall binding. Antimicrob Agents Chemother.

[10] Degli Esposti M (1998). Inhibitors of NADH-ubiquinone reductase: an overview. Biochim Biophys Acta.

[11] Calahorra M, Sánchez NS, Peña A (2012). Characterization of glycolytic metabolism and ion transport of Candida albicans. Yeast.

[12] Noble SM, French S, Kohn LA, Chen V, Johnson AD (2010). Systematic screens of a Candida albicans homozygous deletion library decouple morphogenetic switching and pathogenicity. Nat Genet.

[13] Li D, She X, Calderone R (2016). Functional diversity of complex I subunits in Candida albicans mitochondria. Curr Genet.

[14] She X (2018). A mitochondrial proteomics view of complex I deficiency in Candida albicans. Mitochondrion.

[15] Sun N (2013). Azole susceptibility and transcriptome profiling in Candida albicans mitochondrial electron transport chain complex I mutants. Antimicrob Agents Chemother.

[16] Barrientos A (2002). *In vivo* and in organello assessment of OXPHOS activities. Methods.

[17] Veiga A, Arrabaça JD, Loureiro-Dias MC (2003). Cyanide-resistant respiration, a very frequent metabolic pathway in yeasts. FEMS Yeast Res.

[18] Kerscher SJ (2000). Diversity and origin of alternative NADH:ubiquinone oxidoreductases. Biochim Biophys Acta.

[19] Li D (2011). Enzymatic dysfunction of mitochondrial complex I of the Candida albicans goa1 mutant is associated with increased reactive oxidants and cell death. Eukaryot Cell.

[20] Helmerhorst EJ, Murphy MP, Troxler RF, Oppenheim FG (2002). Characterization of the mitochondrial respiratory pathways in Candida albicans. Biochim Biophys Acta.

[21] Brandt U (2006). Energy converting NADH:quinone oxidoreductase (complex I). Annu Rev Biochem.

[22] Luttik MA (1998). The Saccharomyces cerevisiae NDE1 and NDE2 genes encode separate mitochondrial NADH dehydrogenases catalyzing the oxidation of cytosolic NADH. J Biol Chem.

[23] Hirst J (2013). Mitochondrial complex I. Annu Rev Biochem.

[24] Murphy MP (2009). How mitochondria produce reactive oxygen species. Biochem J.

[25] Marnett LJ (1999). Lipid peroxidation-DNA damage by malondialdehyde. Mutat Res.

[26] Nimse SB, Pal D (2015). Free radicals, natural antioxidants, and their reaction mechanisms. RSC Advances.

[27] Ansari MA, Fatima Z, Hameed S (2016). Antifungal Action of Methylene Blue Involves Mitochondrial Dysfunction and Disruption of Redox and Membrane Homeostasis in C. albicans. Open Microbiol J.

[28] Bondaryk M, Staniszewska M, Zielińska P, Urbańczyk-Lipkowska Z (2017). Natural Antimicrobial Peptides as Inspiration for Design of a New Generation Antifungal Compounds. Journal of Fungi.

[29] Puri S, Edgerton M (2014). How does it kill?: understanding the candidacidal mechanism of salivary histatin 5. Eukaryot Cell.

[30] Gyurko C, Lendenmann U, Troxler RF, Oppenheim FG (2000). Candida albicans mutants deficient in respiration are resistant to the small cationic salivary antimicrobial peptide histatin 5. Antimicrob Agents Chemother.

[31] Helmerhorst EJ (1999). The cellular target of histatin 5 on Candida albicans is the energized mitochondrion. J Biol Chem.

[32] Helmerhorst EJ, Troxler RF, Oppenheim FG (2001). The human salivary peptide histatin 5 exerts its antifungal activity through the formation of reactive oxygen species. Proc Natl Acad Sci USA.

[33] Petruzzelli R (2003). Respiratory inhibition of isolated mammalian mitochondria by salivary antifungal peptide histatin-5. Biochem Biophys Res Commun.

[34] Okun JG, Lummen P, Brandt U (1999). Three classes of inhibitors share a common binding domain in mitochondrial complex I (NADH:ubiquinone oxidoreductase). J Biol Chem.

[35] Kang PT, Yun J, Kaumaya PPT, Chen Y-R (2011). Design and use of peptide-based antibodies decreasing superoxide production by mitochondrial complex I and complex II. Biopolymers.

[36] Horgan DJ, Singer TP, Casida JE (1968). Studies on the respiratory chain-linked reduced nicotinamide adenine dinucleotide dehydrogenase. 13. Binding sites of rotenone, piericidin A, and amytal in the respiratory chain. J Biol Chem.

[37] Zharova TV, Vinogradov AD (1997). A competitive inhibition of the mitochondrial NADH-ubiquinone oxidoreductase (complex I) by ADP-ribose. Biochim Biophys Acta.

[38] Kean EA, Gutman M, Singer TP (1971). Studies on the respiratory chain-linked nicotinamide adenine dinucleotide dehydrogenase. XXII. Rhein, a competitive inhibitor of the dehydrogenase. J Biol Chem.

[39] Kotlyar AB, Karliner JS, Cecchini G (2005). A novel strong competitive inhibitor of complex I. FEBS Lett.

[40] Brown AJP, Brown GD, Netea MG, Gow NAR (2014). Metabolism impacts upon Candida immunogenicity and pathogenicity at multiple levels. Trends Microbiol.

[41] Li D, Calderone R (2017). Exploiting mitochondria as targets for the development of new antifungals. Virulence.

[42] Shingu-Vazquez M, Traven A (2011). Mitochondria and fungal pathogenesis: drug tolerance, virulence, and potential for antifungal therapy. Eukaryot Cell.

[43] McLellan CA (2018). Inhibiting mitochondrial phosphate transport as an unexploited antifungal strategy. Nat Chem Biol.

[44] Li S-X (2017). Mitochondrial Complex V α Subunit Is Critical for Pathogenicity through Modulating Multiple Virulence Properties. Front Microbiol.

[45] Calderone R, Li D, Traven A (2015). System-level impact of mitochondria on fungal virulence: to metabolism and beyond. FEMS Yeast Res.

[46] Lohse MB, Gulati M, Johnson AD, Nobile CJ (2018). Development and regulation of single- and multi-species Candida albicans biofilms. Nat Rev Microbiol.

[47] Höfs S, Mogavero S, Hube B (2016). Interaction of Candida albicans with host cells: virulence factors, host defense, escape strategies, and the microbiota. J Microbiol.

[48] Bambach A (2009). Goa1p of Candida albicans localizes to the mitochondria during stress and is required for mitochondrial function and virulence. Eukaryot Cell.

[49] She X (2015). Fungal-specific subunits of the Candida albicans mitochondrial complex I drive diverse cell functions including cell wall synthesis. Cell Microbiol.

