# Supplementary material for: Novel mitochondrial complex I-inhibiting peptides restrain NADH dehydrogenase activity
Source: Sci Rep. 2019 Sep 23;9:13694. doi: 10.1038/s41598-019-50114-2 (PMC6757105; doi:10.1038/s41598-019-50114-2)
Supplement: Supplementary file 1 — Supplementary Dataset [file 41598_2019_50114_MOESM1_ESM.pdf]

Novel mitochondrial complex I-inhibiting peptides restrain NADH dehydrogenase activity

Yao-Peng Xue[1], Mou-Chieh Kao[2,3*] and Chung-Yu Lan,[1, 3*]

Institute of Molecular and Cellular Biology[1], Institute of Molecular Medicine[2] and Department of Life Science[3]

National Tsing Hua University, No. 101, Section 2, Kuang-Fu Road, Hsinchu 30013, Taiwan, ROC

Supplementary Information

Supplementary Fig. 1

a)

b)

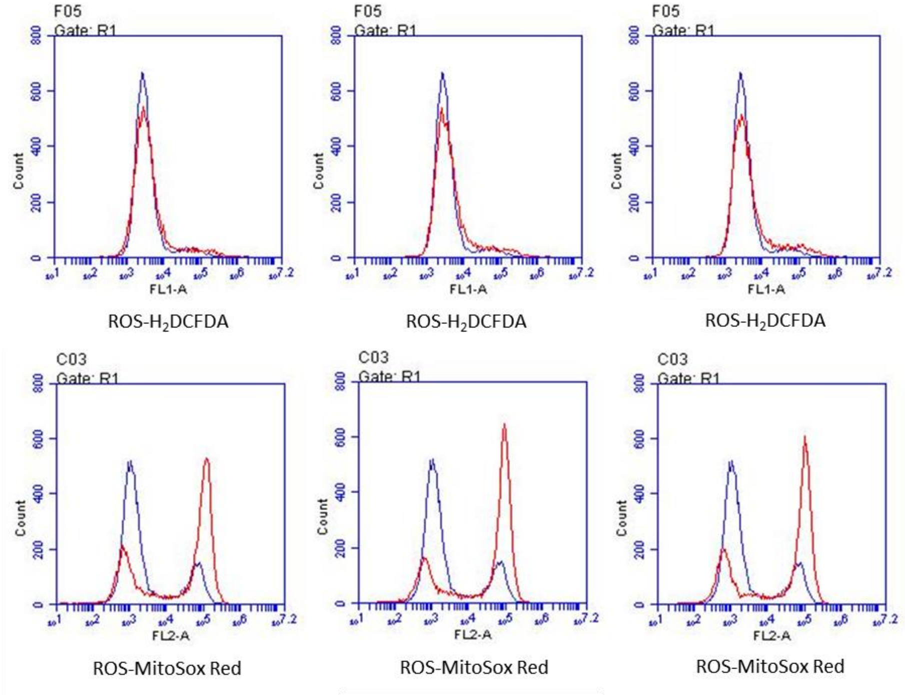

Supplementary Figure 1. The intracellular ROS was detected by H_2DCFDA (**a**) and MitoSOX Red (**b**). The AMPs treated cells (red line) have an increase in fluorescence ROS intensity compared to cells without any treatment (blue line). Cells were treated with a sublethal dose of AMPs at 37°C for 1 h. From left to right is the result of P-113, P-113Du and P-113 Tri. The results shown are from one of the three independent experiments.

Supplementary Fig. 2

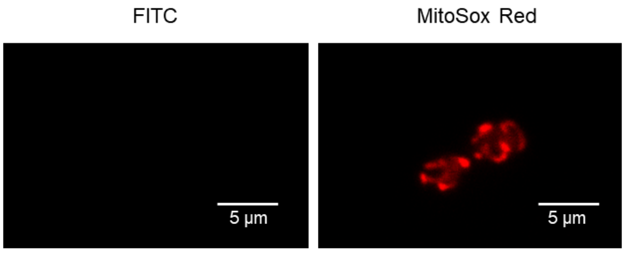

Supplementary Figure 2. The treatment-free control of *C. albicans* fluorescent images. Cells were harvested and washed by 12.5 mM sodium acetate. Mitochondria of *C. albicans* were localized by MitoSOX Red (5μM) at 30°C for 30 min. The result was imaged by Zeiss Axio fluorescence microscope.

Supplementary Fig. 3

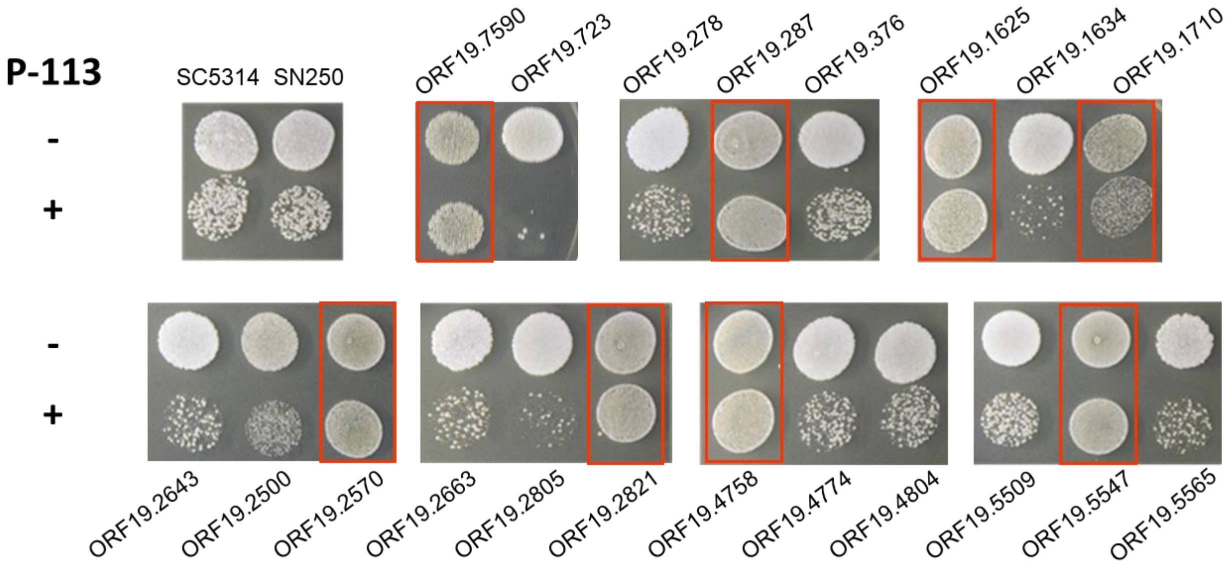

Supplementary Figure 3. Mutant library screening for P-113 resistant strains. Mutants and wild type (SN250 and SC5314) strains were treated with or without P-113 at 37°C for 1 h and spotted on YPD agar. Results were compared between each cell with or without P-113 treatment. The systematic name of each mutants were indicated.

Supplementary Fig. 4

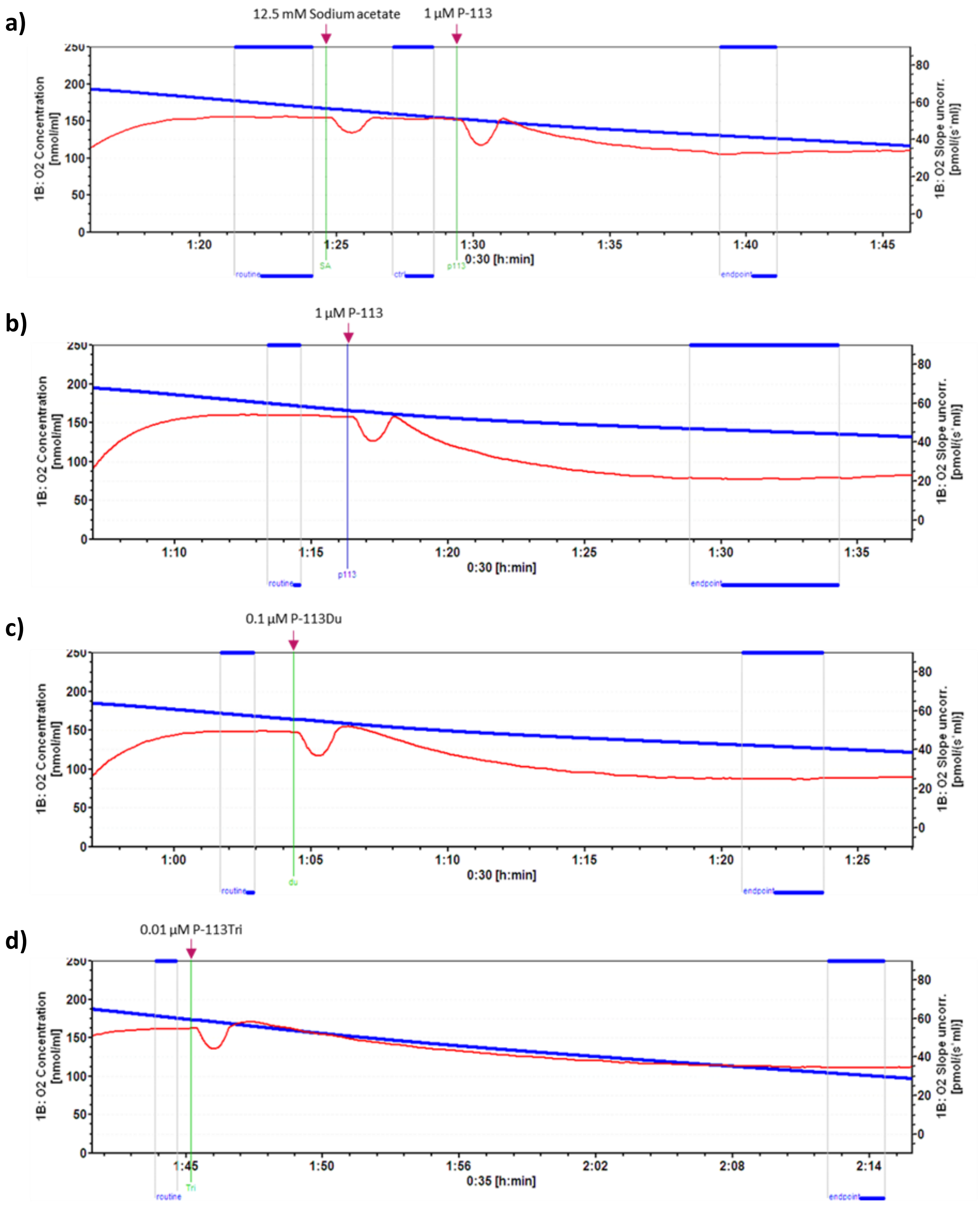

Supplementary Figure 4. Oxygen consumption rates are inhibited by AMPs. The assays were performed using an Oxygraph-2k respirometer and the diagrams show the dynamical oxygen concentration (blue) within the implementation camber and the oxygen consumption rate (red) of cells. Cells were treated with (**a**) 12.5 mM sodium acetate (SA) as the negative control, and followed by adding 1 µM P-113. In other assays, cells were only treated with (**b**) 1 µM P-113, (**c**) 0.1 µM P-113Du, (**d**) 0.01 µM P-113Tri, respectively. All experiments were repeated independently at least three times and representative plots from three experiments with similar results are shown.

Supplementary Fig. 5

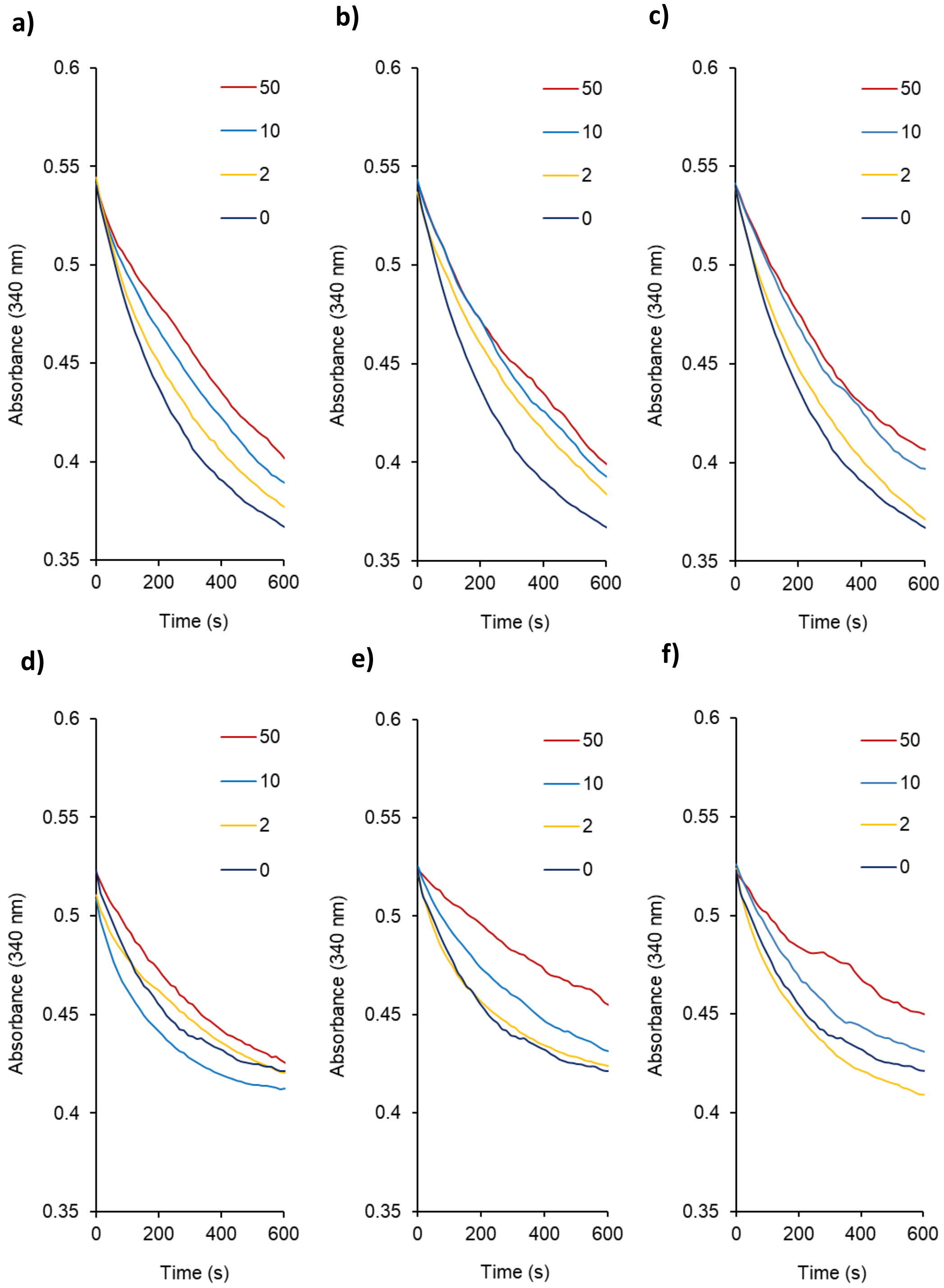

Supplementary Figure 5. Mitochondrial complex I and NADH dehydrogenase inhibiting activity of AMPs. Mitochondria were treated by AMPs at 37°C for 2 h and detected at 340 nm. Diagrams (**a-c**) used NADH as the electron donor and CoQ1 as the electron acceptor. (**a**) P-113, (**b**) P-113Du, and (**c**) P-113Tri have shown concentration-dependent activity. Results (**d-f**) used NADH as the electron donor and ferricyanide as the electron acceptor. (**d**)P-113, (**e**) P-113Du, and (**f**) P-113Tri have shown concentration-correlated activity against NADH dehydrogenase on complex I. The results are from one of three independent experiments.
